# EHMTI-0286. Hemicrania epileptica: case report

**DOI:** 10.1186/1129-2377-15-S1-C61

**Published:** 2014-09-18

**Authors:** F Traverso, M Fasciglione, F Alberti

**Affiliations:** 1 Neurology, ASL 1 IMPERIESE, Sanremo, Italy; 2 Radiolology, ASL 1 IMPERIESE, Sanremo, Italy

## 

Headache occurring during partial seizure, ipsilateral to the epileptic discharge, and remitting immediately soon after the seizures has terminated, is mentioned in IHS Classification 3rd edition (7.6.1). We here report the case of a misunderstood partial epilepsy, headache being considered the main problem. A fifty years old woman presented with a ten days history of stabbing left lateralized headache. The attacks were short-lasting (<3 minutes), at least forty or more every day, neuralgiform without conjunctival injection or tearing, but always associated with elementary hallucination (a bright light) in the right hemifield. She had a a history of very severe head trauma at age twenty-one, with left hematoma evacuation, followed by hydrocephalus, installation of ventriculo-peritoneal shunt complicated by meningitis. She had the same headache attacks at age thirty for a period of three months. She took several analgesic preparation every day with benefit on headache but not on right sided hallucinations. EEG showed left spikes discharges on the left temporo-occipital regions occurring during the headache with hallucinations attacks and remitting immediately after the seizures has terminated. CT demonstrated left porencephalic cavity, ex vacuo dilatation of left lateral ventricle, left temporo-parietal fracture, ventricolo-peritoneal catheter in right lateral ventricle. The seizures remitted with antiepileptic therapy (levetiracetam 500 mg twice daily). Our case report demostrates the importance of considering the differential diagnosis of epilepsy in stabbing headache even when analgesic sensitive.

No conflict of interest.

